# Characterization and Expression of KT/HAK/KUP Transporter Family Genes in Willow under Potassium Deficiency, Drought, and Salt Stresses

**DOI:** 10.1155/2020/2690760

**Published:** 2020-06-09

**Authors:** Meixia Liang, Yachao Gao, Tingting Mao, Xiaoyan Zhang, Shaoying Zhang, Hongxia Zhang, Zhizhong Song

**Affiliations:** ^1^College of Agriculture, Ludong University, Yantai 264000, China; ^2^Key Laboratory of Molecular Module-Based Breeding of High Yield and Abiotic Resistant Plants in Universities of Shandong (Ludong University), Yantai 264000, China; ^3^College of Agriculture, Inner Mongolia Agricultural University, Huhhot 010018, China; ^4^College of Forest, Nanjing Forest University, Nanjing 214008, China

## Abstract

The K^+^ transporter/high-affinity K^+^/K^+^ uptake (KT/HAK/KUP) transporters dominate K^+^ uptake, transport, and allocation that play a pivotal role in mineral homeostasis and plant adaptation to adverse abiotic stresses. However, molecular mechanisms towards K^+^ nutrition in forest trees are extremely rare, especially in willow. In this study, we identified 22 KT/HAK/KUP transporter genes in purple osier willow (designated as *SpuHAK1* to *SpuHAK22*) and examined their expression under K^+^ deficiency, drought, and salt stress conditions. Both transcriptomic and quantitative real-time PCR (qRT-PCR) analyses demonstrated that *SpuHAKs* were predominantly expressed in stems, and the expression levels of *SpuHAK1*, *SpuHAK2*, *SpuHAK3*, *SpuHAK7*, and *SpuHAK8* were higher at the whole plant level, whereas *SpuHAK9*, *SpuHAK11*, *SpuHAK20*, and *SpuHAK22* were hardly detected in tested tissues. In addition, both K^+^ deficiency and salt stress decreased the tissue K^+^ content, while drought increased the tissue K^+^ content in purple osier plant. Moreover, *SpuHAK* genes were differentially responsive to K^+^ deficiency, drought, and salt stresses in roots. K^+^ deficiency and salt stress mainly enhanced the expression level of responsive *SpuHAK* genes. Fifteen putative *cis*-acting regulatory elements, including the stress response, hormone response, circadian regulation, and nutrition and development, were identified in the promoter region of *SpuHAK* genes. Our findings provide a foundation for further functional characterization of KT/HAK/KUP transporters in forest trees and may be useful for breeding willow rootstocks that utilize potassium more efficiently.

## 1. Introduction

As one of the most abundant cations in plant cells, potassium (K^+^) is involved in many physiological and metabolic processes, such as stomatal movement, photosynthesis, respiration, cellular osmoregulation, and enzyme activation [1–3]. Application of K^+^ fertilizer favorably improved leaf growth [4, 5], flowering [6], wood quality, and yield [7–9]. However, mechanisms underlying K^+^ nutrition in perennial forest trees are limited [10].

Plants need to uptake an optimal amount of K^+^ via high-affinity K^+^ transporter uptake system in roots from the soil, to maintain normal growth [3, 11, 12]. In particular, K^+^ transporters can be divided into four families: KT/HAK/KUP, Trk/HKT, CHX, and KEA [1, 13], which play an important role in improving plant tolerance to different abiotic stresses such as drought [14–16], salt [17–21], and heavy metal stresses [22, 23]. Notably, KT/HAK/KUP transporters are one of the largest K^+^ transporter families, which function in acquiring K^+^, catalyzing K^+^ uptake across a wide range of external concentrations, mediating K^+^ movement within the plant as well as its efflux into the environment, and maintaining ion homeostasis in plants [1, 3, 13, 24].

Recent years, a number of KT/HAK/KUP transporters have been identified in diverse organisms, especially in annual plants, including *Arabidopsis* [25–27], barley [28], rice [29–31], maize [32], alligator weed [21, 33], and tomato [34], and recently in fruit trees of peach [35, 36] and pear [37, 38]. The possible functions of several plant KT/HAK/KUP family members have been characterized by T-DNA insertion mutants [18, 27, 31, 39], overexpression in model plants [18, 21], and heterologous expression in bacteria mutant [25, 35] or yeast [12, 17, 39]. Although there are more than 650 species of Salicaceae plants in the world, the functions of KT/HAK/KUP transporters in Salicaceae are still unknown, just observed in the gene identification in poplar [10]. In particular, the plant KT/HAK/KUP family transporters are divided into three to four subgroups, including Group I, II, III, and IV, during the long evolution [30, 32, 34, 35, 37, 38]. The knowledge on K^+^ uptake and transport in model plants has provided some insights into the investigation of their roles in forest trees.

As one of the most popular diploid willow plants, purple osier willow (*Salix purpurea*) plays an important role in soil and water conservation, shelter forest, and biomass energy, and its genome has been successfully sequenced [40–42]. The molecular basis and mechanisms towards K^+^ nutrition and homeostasis in strawberry are essentially unknown. In this study, we identified 22 KT/HAK/KUP family transporter genes (*SpuHAK*s) in diploid purple osier willow and analyzed their expression profiles under both normal and K^+^ deficiency conditions, which provided gene resources for revealing the mechanism of K^+^ uptake, transport, and distribution in woody trees and provided a theoretical basis for the control of K^+^ fertilizer application in woody trees and efficient genetic manipulation and breeding of willow plants.

## 2. Materials and Methods

### 2.1. Plant Material and Growth Condition

The 1-year old female purple osier plants (a gift from Nanjing Forest University in Nanjing, China) were obtained by cutting asexual cloning and used throughout this study. Plants were grown in a growth chamber with 12 h light at 25°C followed by 12 h dark at 20°C (with 60% relative humidity). Leaf, stem, root, full blooming flower, young fruit (with a diameter of 0.5 cm in green colour), and mature fruit (with a full size in red color) tissues of Yellow Wonder 5AF8 were collected from the same plant on April 15th, 2018 and frozen immediately in liquid nitrogen for further RNA extraction and quantitative real-time PCR analyses.

Plants were grown in the control conditions (half-strength MS basal medium [43], supplemented with 1 mM K^+^, 2% sucrose, 1% agar, and 0.5 g L^−1^ of MES, pH 5.7) in a growth chamber for 2 weeks, and then transferred to the1/2MS solution in plastic containers. The nutrient solution was changed every other day. For K^+^ deficiency treatment, K^+^ was omitted from the 1/2MS medium by adding equal molar Na^+^ to keep the concentration of N stabilization. In drought treatments, plants were exposed to 1/2MS supplemented with 15% (*w*/*v*) PEG6000. In salt treatments, plants were suffered to 1/2MS supplemented with 150 mmol·L^−1^ NaCl. Plants were exposed to K^+^ deficiency treatment for 72 h, and then suffered to qRT-PCR and K^+^ content determination. K^+^ content was measured as described by Song and Su [33]; plant samples were dried and digested using the HNO_3_-HCLO_4_ method and subjected to ICP-AES (IRIS Advantage, Thermo Electron, Waltham, MA, USA).

### 2.2. Identification of SpuHAK Genes in Purple Osier Willow

Genome information of purple osier willow was screened from The Plant Genomics Resource from Phytozome 12 (https://phytozome.jgi.doe.gov/pz/portal.html). To obtain all the KT/HAK/KUP family genes in purple osier willow, BLAST searches against the genome database were carried out with the full-length of 13 Arabidopsis KT/HAK/KUP protein sequences as references. The amino acid sequences of proteins codified by candidate purple osier willow KT/HAK/KUP genes were verified using the InterProScan 4.8 (http://www.ebi.ac.uk/Tools/pfa/iprscan/) and Pfam (http://pfam.xfam.org), to confirm the existence of K^+^ transporter (PF02705) domains. Candidate genes without K^+^ transporter domains were removed. Intron numbers were gathered on Phytozome Genomics Resources. Physicochemical properties of KT/HAK/KUP transporters were calculated using the ProtParam tool (http://web.expasy.org/protparam/), including amino acid length, theoretical isoelectric point (P*I*), molecular weight, instability index, aliphatic index, and grand average of hydropathicity (GRAVY). Subcellular localization prediction was performed on the PSORT Server (https://www.genscript.com/psort.html). Putative *cis*-acting regulatory elements were predicted on the PlantCARE (http://bioinformatics.psb.ugent.be/webtools/plantcare/html/) online server.

### 2.3. Motif Display and Phylogenetic Analysis of SpuHAK Transporters

The full-length KT/HAK/KUP protein sequences of purple osier willow, Arabidopsis, rice, strawberry, peach, pear, and poplar were downloaded from the Phytozome Genomics Resources (purple osier willow), Arabidopsis Information Resource (TAIR) (http://www.arabidopsis.org), the Rice Genome Annotation Project (http://rice.plantbiology.msu.edu/), Strawberry Genome Database from GDR (https://www.rosaceae.org), Peach Genome Database (Assembly v2.0) from GDR (https://www.rosaceae.org), Pear Genome Project (http://peargenome.njau.edu.cn/), and Poplar Genome Database JGI v2.0 (http://www.plantgdb.org/PtGDB/), respectively. A phylogenetic tree was constructed by multiple alignment of KT/HAK/KUP proteins in purple osier willow Arabidopsis, rice, strawberry, peach, pear, and poplar using ClustalX2.1 and MEGA7.0 software, based on 1000 bootstrap replicates neighbor-joining method [44] (Tamura et al. 2007).

### 2.4. Gene Expression Pattern Prediction

The transcriptomic data for purple osier willow were downloaded from Phytozome Genomics Resources. The transcriptomic data (RPKM) were calculated using a log2 scale, and the heatmap was plotted using HemI software according to the method described by Deng et al. [45].

### 2.5. RNA Extraction and Quantitative Real-Time PCR Assays

Total RNA was extracted using MiniBEST Plant RNA Extraction Kit (TaKaRa, Dalian, China) and reverse transcribed into cDNA using the PrimeScript™ RT reagent Kit (TaKaRa, Dalian, China). Specific primers for *SpuHAK* transporter genes and *Ubiquitin* control gene were designed using NCBI/Primer-BLAST online server. Primer sequences were listed in Supplementary Table 1. Quantitative real-time RT-PCR (qRT-PCR) was carried out on 7500 Real-Time PCR System (Applied Biosystems, New York, USA), using SYBR Premix Ex Taq reaction kit (TaKaRa, Dalian, China), as described by Song et al. [21, 35]. To calculate RT-qPCR efficiency and the starting template concentration for each sample, the linear regression of the log (fluorescence) per cycle number data was used according to the description of Deng et al. [45]. The relative expression levels of the *SpuHAK* genes were presented after normalization to the internal control *Ubiquitin* from three independent biological repeats.

### 2.6. Statistical Analysis

All data were statistically analyzed using independent samples *t* test in SPSS 13.0 software (SPSS Chicago, Illinois, USA). Asterisks indicate statistical differences between plants under control and stress treatment (^∗^*P* < 0.05, ^∗∗^*P* < 0.01, independent-samples *t* test). Data were compared between plants under control and stress treatment. Details are described in figure legends. Graphs were produced using Origin 8.0 software.

## 3. Results

### 3.1. Identification of SpuHAK Genes in Purple Osier Willow

By BLAST searching of the Phytozome Genomics Resources (purple osier willow), 22 putative strawberry *SpuHAK* genes were identified, which were entitled as *SpuHAK1* to *SpuHAK22*. Protein domain verification analyses showed that all of them contain the K^+^ transporter transmembrane domain (PF02705). Except *SpuHAK14* and *16* which are still unclear, all the other *SpuHAK* genes were distributed on 9 distinct chromosomes, in which 6 genes on the 3rd chromosome and 5 genes on the 1st chromosome. All *SpuHAK* genes possess 5 to 9 introns that varied distinctly in length. Detailed information about these *SpuHAK* genes, including gene ID, gene location, CDS (coding sequence) length, peptide length, and intron number, is provided in Table 1.

The properties of SpuHAK proteins were also analyzed. The molecular weight of these predicted proteins range from 64.65 to 95.39 kDa correspondingly (Table 2). The amino acid sequences of s SpuHAK proteins share an overall identity of 51.09% (data not shown). Instability index assays implicated that 15 of the 22 SpuHAK proteins were stable proteins, whereas the remaining 7 members were unstable proteins (Table 2). According to the value of theoretical P*I*, 17 of the 22 SpuHAK proteins were alkalescent, and the remaining 5 members were acidic (Table 2). Moreover, the GRAVY index indicated that all of the SpuHAK proteins in purple osier willow are hydrophobic proteins with positive values, and aliphatic index analyses illustrated that all SpuHAK proteins had high values above 100, which supports the predication that SpuHAKs are hydrophilic proteins (Table 2).

### 3.2. Phylogenetic and Protein Motif Analysis of SpuHAK Proteins

**Figure 1:**
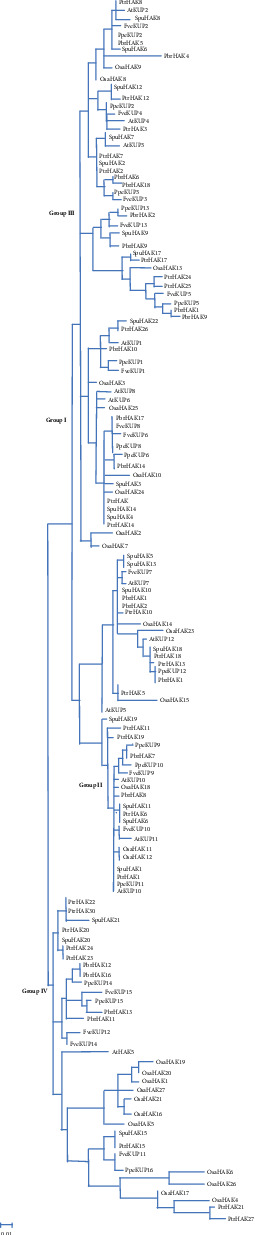
Phylogenetic tree of the KT/HAK/KUP family proteins from different plants. A Maximum Likelihood (ML) tree was constructed by multiple alignments of KT/HAK/KUP proteins in purple osier willow, *Arabidopsis*, rice, strawberry, pear, peach, and poplar using ClustalX2.1 and MEGA7.0 software. The tree was based on 1000 bootstrap replicates neighbor-joining method. The plant KT/HAK/KUP family members were distributed on four subgroups (Groups I-IV, marked in blue), and the purple osier willow KT/HAK/KUP proteins were labeled with a small red circle.

To confirm the evolutionary relationships of SpuHAK proteins, a Maximum Likelihood (ML) phylogenetic tree was generated based on the alignment of the KT/HAK/KUP amino acid sequences in purple osier willow, *Arabidopsis*, rice, strawberry, peach, pear, and poplar. All plant KT/HAK/KUP transporters were classified into 4 major groups (I-IV, Figure 1). The SpuHAK proteins were randomly distributed in Groups I-IV, each with 8, 4, 7, and 3 members, respectively (Figure 1 and Table 2). Purple osier willow and poplar belong to the same family of *Salicaceae*; all of the 22 SpuHAK members were closely clustered with the corresponding poplar orthologs in the phylogenetic tree, one to one or two to one, respectively (Figure 1). Moreover, all Roseaceae orthologs from strawberry, peach, and pear have the closest genetic relationship (Figure 1).

### 3.3. Subcellular Localization Prediction

Subcellular localization prediction showed that all SpuHAK proteins were mainly localized in the plasma membrane, followed by mitochondrial inner membrane except for SpuHAK5, SpuHAK6, and SpuHAK11 (Table 3). In addition, SpuHAK proteins were also observed in the vesicles of the secretory system, vacuole membrane, and nucleus, individually. Notably, 9 transporters (SpuHAK1, SpuHAK3, SpuHAK4, SpuHAK8, SpuHAK13, SpuHAK14, SpuHAK15, SpuHAK17, and SpuHAK18) showed similar subcellular localization patterns, while 3 members of SpuHAK5, SpuHAK6, and SpuHAK11, 3 members of SpuHAK10, SpuHAK18, and SpuHAK21, 2 members of SpuHAK2 and SpuHAK9, and 2 members of SpuHAK19 and SpuHAK22 possessed the same localization patterns, respectively (Table 3).

### 3.4. The *Cis*-Acting Regulatory Elements in the Promoter Regions of SpuHAK Genes in Purple Osier Willow

Promoter regions of the *SpuHAK* family genes were obtained from the Phytozome Genome Database via retrieving 2 kb range genomic DNA sequences upstream of the translation start sites of the *SpuHAK* family genes. Prediction showed that at least 13 kinds of *cis*-acting regulatory elements were observed in the promoter regions of *SpuHAK* genes (Table 4), which were involved in abiotic stress response (light, drought inducibility, low temperature, anaerobic induction and defense, and stress), hormone response (salicylic acid, gibberellin, methyl jasmonate, abscisic acid, and auxin), circadian regulation, and nutrition and development (meristem expression and endosperm expression). In particular, two abiotic stress response regulatory elements (light response and anaerobic induction) were detected in all *SpuHAK* family genes, while the other *cis*-acting regulatory elements were found in distinct *SpuHAK* genes with different numbers (Table 4).

### 3.5. Transcriptomic Expression Profiles of SpuHAK in Purple Osier Willow

**Figure 2:**
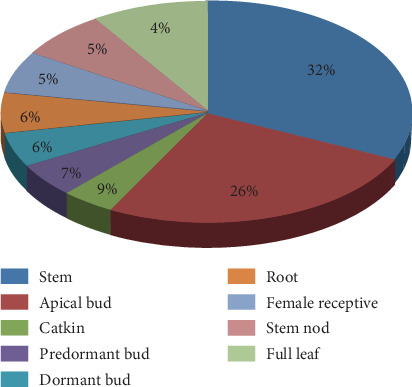
General expression pattern of *SpuHAK*s in different tissues/organs of purple osier willow. The expression levels (RPKM) of *SpuHAK*s were directly downloaded from Phytozome Genomic Resources (purple osier willow).

To gain insights into the possible functions of *SpuHAK* genes during the willow growth and development, the transcriptomic data during purple osier willow development were obtained from Phytozome online database. In general, the percentages of *SpuHAK* gene expression in different tissues and organs are 32% in stem, 26% in the apical bud, and 9% in catkin, followed by 7% in predormant bud, 6% in dormant bud, 6% in root, 5% in female receptive, 5% in stem nod, and 4% in full leaf (Figure 2).

**Figure 3:**
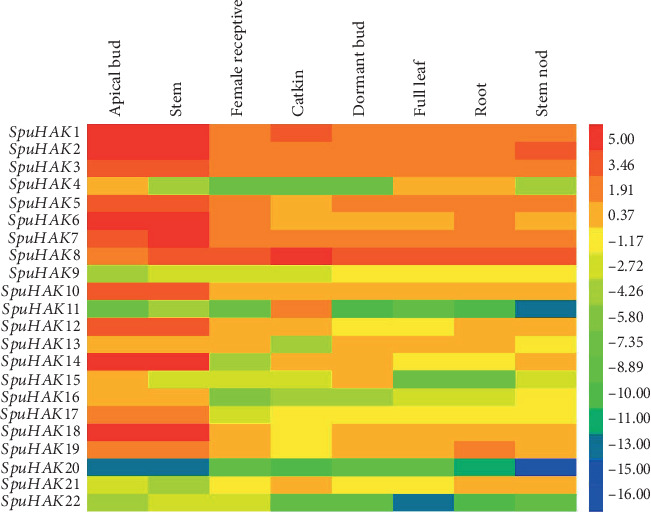
Heatmap of transcriptomic expression profiles of *SpuHAK*s in different tissues/organs of purple osier willow. The expression levels (RPKM) of *SpuHAK*s were directly downloaded from Phytozome Genomic Resources (purple osier willow) and plotted as a log2 scale. Red and blue boxes indicate high and low expression levels, respectively.

In particular, *SpuHAK* genes were expressed differently in distinct tissues and organs (Figure 3). The expression levels of *SpuHAK1*, *SpuHAK2*, *SpuHAK3*, *SpuHAK7*, and *SpuHAK8* were higher at the whole plant level, whereas *SpuHAK9*, *SpuHAK11*, *SpuHAK20*, and *SpuHAK22* were extremely low in tested tissues (Figure 3).

### 3.6. qRT-PCR Determination of SpuHAK Genes in Purple Osier

**Figure 4:**
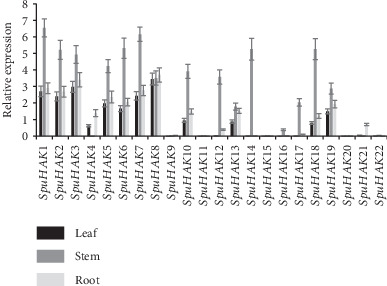
Quantitative real time-PCR analysis of *SpuHAKs* in purple osier willow. 1-year old female purple osier plants were obtained by cutting asexual cloning. Plants were grown in 1/2MS liquid medium in a growth chamber with 12 h light at 25°C followed by 12 h dark at 20°C (with 60% relative humidity). Leaf, stem, and root tissues of Yellow Wonder 5AF8 were collected from the same plant on April 15th, 2018, and frozen immediately in liquid nitrogen for RNA extraction and quantitative real-time PCR.

We further performed qRT-PCR to determine the expression profiles of *SpuHAK* genes in different tissues of 1-year old female purple osier. Results showed that *SpuHAK* genes were unevenly expressed in the tested organs, including leaves, stems, and roots (Figure 4). The expression levels of *SpuHAK1*, *SpuHAK2*, *SpuHAK3*, *SpuHAK5*, *SpuHAK6*, *SpuHAK7*, and *SpuHAK8* was higher than that of the other genes, at the whole plant level, whereas *SpuHAK9*, *SpuHAK11*, *SpuHAK15*, *SpuHAK20*, and *SpuHAK22* were extremely low in tested tissues (Figure 4), which was exactly consistent with that of the transcriptomic expression profiles (Figure 3). In details, 14 of the 22 *SpuHAK* genes (i.e., *SpuHAK1*, *SpuHAK2*, *SpuHAK3*, *SpuHAK5*, *SpuHAK6*, *SpuHAK7*, *SpuHAK10*, *SpuHAK12*, *SpuHAK13*, *SpuKU14*, *SpuHAK16*, *SpuHAK17*, *SpuHAK18*, and *SpuHAK19*) were mainly expressed in stems, more than in the other tissues, and *SpuHAK4* and *SpuHAK21* were mainly expressed in roots, whereas *SpuHAK8* was evenly expressed throughout the whole plant (Figure 4).

### 3.7. SpuHAK Gene Expression under K^+^ Deficiency, Drought, and Salt Conditions

**Figure 5:**
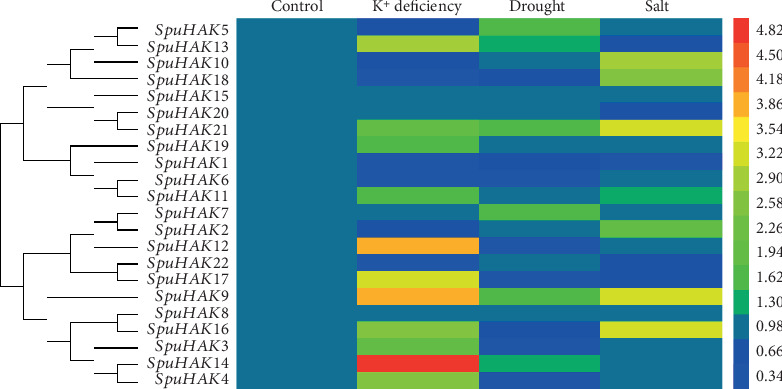
Expression changes of *SpuHAK*s under K^+^ deficiency, drought, and salt stress treatments. 1-year-old purple osier plants were subjected to K^+^ deficiency, drought (15% (*w*/*v*) PEG6000), and salt (150 mmol·L^−1^ NaCl) treatments for 72 h before examination. Each *SpuHAK* gene was analyzed in leaves, stems, and roots. The relative expression level of genes was presented after normalization to the internal control from three independent biological repeats.

The transcript level changes after stress treatment is an important factor for a transporter functioning as a high-affinity K^+^ uptake in the root epidermis [46]. To investigate the role of *SpuHAK* in uptaking and maintaining K^+^ homeostasis in willow, especially under adverse conditions, we analyzed the expression profiles of *SpuHAK* genes in roots of purple osier under K^+^ deficiency, drought, and salt stresses. Results showed that the expression of *SpuHAKs* was differentially affected by K^+^ deficiency, drought, and salt stresses in roots (Figure 5). In particular, 13 out of 22 *SpuHAKs* were responsive to K^+^ deficiency, in which 10 genes (*SpuHAK3*, *SpuHAK4*, *SpuHAK9*, *SpuHAK12*, *SpuKU13*, *SpuHAK14*, *SpuHAK16*, *SpuHAK17*, *SpuHAK19*, and *SpuHAK21*) were significantly enhanced, and the remaining 3 genes (*SpuHAK1*, *SpuHAK6*, and *SpuHAK18*) were dramatically reduced (Figure 5). Expression of 6 genes were affected by drought stress, in which *SpuHAK5*, *SpuHAK7*, and *SpuHAK21* were increased and *SpuHAK3*, *SpuHAK4*, and *SpuHAK12* were decreased (Figure 5). Expression of 8 genes was altered by salt stress, which were all significantly induced except for *SpuHAK1* that was reduced (Figure 5). Notably, *SpuHAK8* was evenly expressed in tested tissues of purple osier at a moderate level and had no response to any treatment. Expression of *SpuHAK15* and *SpuHAK20* were extremely low in purple osier and also had no effect to any treatment (Figure 5).

**Figure 6:**
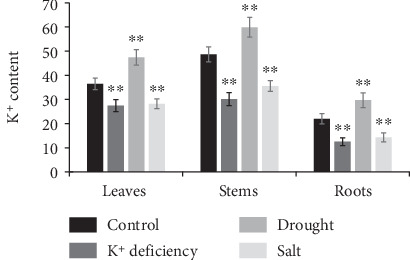
Determination of K^+^ content in different tissues of purple osier plants.1-year-old purple osier plants were subjected to K^+^ deficiency, drought (15% (*w*/*v*) PEG6000), and salt (150 mmol·L^−1^ NaCl) treatments for 72 h, and then digested using the HNO_3_-HCLO_4_ method and determined by ICP-AES. Asterisks indicate statistical differences between plants under control and stress treatment (^∗∗^*P* < 0.01, independent-samples *t* test).

In addition, the tissue K^+^ accumulation was higher in shoots than in roots, and the highest was observed in stems (Figure 6). Both K^+^ deficiency and salt stresses decreased the tissue K^+^ concentration, while drought stress strengthened the tissue K^+^ concentration, in all tested tissues (Figure 6).

## 4. Discussion

KT/HAK/KUP family transporters play a major role in catalyzing K^+^ acquisition and uptake and maintaining plant cation homeostasis, which further contributes to plant growth and development [1, 5, 13, 26]. Genome-wide analysis of the KT/HAK/KUP gene family has been reported in various plants [1, 12]. However, the molecular mechanism of K^+^ absorption in willow has not been mentioned yet.

Purple osier grows fast and has strong adaptability that plays important roles in water and soil conservation, shelter forest, and bioenergy [47–50]. In this present study, we identified and characterized 22 KT/HAK/KUP transporters in purple osier willow. In terms of the woody plants, the number of KT/HAK/KUP transporters genes in purple osier willow is more than that in peach (16) [26], but less than that in poplar (31) [10]. These findings may reflect the fact that purple osier willow has a larger genome of 392 Mbp [40, 41], more than that of peach (230 Mbp) [51], much shorter than that of poplar (520 Mbp) [52]. Although a similar number of genes is similar to that in pear (21) [37, 38], the genome of purple osier willow was much shorter than that of pear (512 Mbp) [53]. Moreover, the phylogenetic tree of KT/HAK/KUP transporters in purple osier willow strictly followed the same distribution into four groups, which is similar to that in rice [30], poplar [10], and pear [37, 38]. In particular, all of KT/HAK/KUP transporters in purple osier were closely clustered with corresponding orthologs of poplar (Figure 1), implying that KT/HAK/KUP family transporters may be highly conserved among Salicaceae plants.

Eukaryotic genes contain exons and introns, which is one of the characteristics of the former to distinguish prokaryotes. Introns play an important role in alternative splicing, and a gene can produce many different proteins. Introns being the essential entities of eukaryotic gene families during the evolution of multiple gene families. In this present study, the intron No. was quite different among *SpuHAK* family genes, and *SpuHAK5* and *SpuHAK10* possess the most (9) introns, while *SpuHAK13* possesses the least (5) introns that varied distinctly in length. These findings may reflect the distinct roles in catalyzing K^+^ acquisition and uptake and maintaining plant cation homeostasis, which still lacks molecular evidences. In addition, the KT/HAK/KUP family transporters have extensive and delicate localizations in plant cells that may share diverse functions or have common and recent evolutionary origins [30, 32, 37]. In purple osier willow, SpuHAK transporters were predicted to mainly localize in the plasma membrane and organelle membranes, including mitochondrion and vacuole. Genomic transcriptomic data indicated that *SpuHAK* genes are differently expressed in distinct tissues or organs of purple osier (Figures 2 and 3). Further, qRT-PCR analysis demonstrated that *SpuHAK* genes were unevenly expressed in tested organs, including leaves, stems, and roots (Figure 4). All these findings indicate that the KT/HAK/KUP family members have extensive and delicate localization and expression patterns in purple, and each gene may have a unique role during distinct biological or developmental processes. Furthermore, it is worth mentioning that a majority of *SpuHAK* genes were highly expressed in stems, more than that in leaves and roots, which was in accordance with the expression profiles of transcriptomic data, implying that these genes are prone to be functional K^+^ transporters, especially during maintaining K^+^ accumulation and homeostasis in willow stems. These findings again support the proposition that K^+^ favorably accumulates in stems that will be further allocated to different parts of the aboveground [26, 33, 38].

In plants, the root system provides sufficient surface area, where K^+^ nutrients need to be transported through the root surface via K^+^ transporters [24, 33]. We found that K^+^ deficiency significantly reduced tissue K^+^ accumulation, which was similar to the previous reports [15, 21, 22, 26, 33, 38]. When suffered of adverse abiotic stresses, plants have to urgently adjust their nutritional status or enhance the internal metabolic systems to maintain basic growth [22, 23, 33, 38]. Undoubtedly, the ubiquitous presence of KT/HAK/KUP transporters in plants implies that they play critical roles in acquiring nutrients and improving plant tolerance to adverse environmental conditions, including K^+^ deficiency, drought, and salt [15–21, 33]. *Cis*-acting regulatory elements, especially of abiotic stress response elements, can control promoter efficiency by combining with key elements in the promoter region and then regulate target gene expression [22, 30, 32]. In this work, at least 5 kinds of abiotic stress-responsive elements, including drought inducibility and defense and stress, were found in *SpuHAK* genes (Table 4). Further qRT-PCR analyses showed that *SpuHAK* genes respond differently to K^+^ deficiency, drought, and salt stresses, implying that they may contribute to stress resistance favorably in willow plants. In details, 13 out of 22 *SpuHAK* genes were responsive to K^+^ deficiency, and 10 genes (in which 5 genes of *SpuHAK3*, *SpuHAK4*, *SpuHAK9*, *SpuHAK14*, and *SpuHAK16* belong to Group I) were significantly upregulated, indicating that these genes are prone to be active in K^+^ uptake or transport in willow roots to maintain “optimal utilization” of K^+^ under limited K^+^ conditions. Again, these findings support the proposition that Group I members exhibit a more specialized K^+^ transporter function during K^+^ deficiency condition [12, 31, 38, 54].

Drought treatment strengthened the K^+^ content especially in aboveground parts, indicating that the water status makes a great contribution to the internal K^+^ concentration. Simultaneously, drought stress stimulated the expressions of 3 genes (SpuHAK5, *SpuHAK7*, and *SpuHAK21*), in which drought inducibility regulatory elements were detected, but reduced the expressions of 3 *SpuHAK* genes, implying that these *SpuHAK* genes may be involved in adaptation to drought stress in quite a complicate way. When plant faced salinity stress, the radius of Na^+^ and K^+^ ions is close and a great amount of Na^+^ may compete with K^+^ that could be preferentially absorbed. Salt treatment greatly decreased the K^+^ content throughout the whole plant and upregulated 7 out of 8 salt-responsive *SpuHAK* genes (Figure 5), suggesting that these SpuHAK transporters may play crucial roles in mobilizing the maximum root uptake and accumulation of external K^+^ to maintain “maximal uptake” of external K^+^ for vital activities under salt stress. Moderate K^+^ transport has to be accumulated and maintained in plants to cope with adverse abiotic stresses by regulating the expression of specific genes [37, 55]. Nonetheless, SpuHAK transporters that respond to such abiotic stresses are likely to function in K^+^ transport, which is indispensible for purple osier willow adaptation to undesired stresses.

## 5. Conclusions

22 KT/HAK/KUP transporter genes were identified and characterized which were predominantly expressed in stems, in purple osier willow. Both K^+^ deficiency and salt stress decreased the tissue K^+^ content, while drought increased the tissue K^+^ content in the purple osier plant. *SpuHAK* genes were unevenly expressed in the tested organs, and K^+^ deficiency and salt stress mainly enhanced the expression of responsive *SpuHAK* genes. Physiological and molecular function determination of *SpuHAK* genes will be further studied.

## Figures and Tables

**Table 1:** Information of the SpuKUP genes in purple osier willow.

Gene	Gene ID	Gene location	CDS (bp)	Peptide length (aa)	Intron no.
*SpuHAK1*	SapurV1A.0324s0140	chr01:8777068..8783359 reverse	2388	795	7
*SpuHAK2*	SapurV1A.0071s0500	chr02:21258203..21262096 reverse	2274	757	8
*SpuHAK3*	SapurV1A.0062s0150	chr10:7832862..7838850 forward	2343	780	7
*SpuHAK4*	SapurV1A.0200s0110	chr08:10272419..10279135 forward	2343	780	7
*SpuHAK5*	SapurV1A.0809s0140	chr03:1699236..1708112 forward	2580	859	9
*SpuHAK6*	SapurV1A.0325s0230	chr03:9740033..9747064 forward	2397	799	7
*SpuHAK7*	SapurV1A.0542s0070	chr14:10944729..10950061 forward	2352	783	8
*SpuHAK8*	SapurV1A.0109s0240	chr13:11331285..11336813 forward	2379	792	8
*SpuHAK9*	SapurV1A.0665s0050	chr15:4488051..4493808 reverse	2373	790	8
*SpuHAK10*	SapurV1A.0344s0140	chr01:16974452..16982662 forward	2265	754	9
*SpuHAK11*	SapurV1A.2895s0010	Scaffold2895:7060..14365 forward	2397	799	7
*SpuHAK12*	SapurV1A.0119s0310	chr03:11725061..11730864 reverse	2331	776	7
*SpuHAK13*	SapurV1A.0952s0110	chr03:1719046..1725662 forward	1737	578	5
*SpuHAK14*	SapurV1A.4781s0010	Scaffold4781:729..7127 forward	2490	829	8
*SpuHAK15*	SapurV1A.0052s0820	chr14:10064169..10070274 forward	2334	777	7
*SpuHAK16*	SapurV1A.1794s0040	Scaffold1794:23249..29732 reverse	2382	793	8
*SpuHAK17*	SapurV1A.0059s0490	chr03:12541705..12546955 reverse	2244	747	8
*SpuHAK18*	SapurV1A.0062s0160	chr10:7824447..7830915 forward	2535	844	8
*SpuHAK19*	SapurV1A.0324s0130	chr01:8767798..8774115 reverse	2373	790	7
*SpuHAK20*	SapurV1A.0444s0160	chr01:4593739..4598741 forward	2466	821	7
*SpuHAK21*	SapurV1A.0444s0170	chr01:4608352..4613238 forward	1860	619	8
*SpuHAK22*	SapurV1A.0042s0410	chr09:5922572..5927384 forward	2463	820	8

**Table 2:** Information of the SpuHAK proteins identified in this work.

Protein	Mw (kDa)	P*I*	Instability index	GRAVY	Aliphatic index	Group
SpuHAK1	89.59	8.55	37.98 (stable)	0.32	106.36	II
SpuHAK2	84.21	9.09	39.41 (stable)	0.50	114.45	IV
SpuHAK3	87.41	8.23	36.48 (stable)	0.34	109.79	I
SpuHAK4	87.18	8.40	39.18 (stable)	0.32	107.83	I
SpuHAK5	95.39	5.82	41.24 (unstable)	0.29	106.07	III
SpuHAK6	90.03	8.83	35.75 (stable)	0.32	106.82	II
SpuHAK7	87.00	9.09	41.96 (stable)	0.46	109.92	IV
SpuHAK8	88.06	6.83	41.47 (unstable)	0.38	107.42	I
SpuHAK9	88.68	8.72	35.24 (stable)	0.27	101.18	I
SpuHAK10	83.85	5.09	41.02 (unstable)	0.38	111.51	III
SpuHAK11	90.01	8.83	35.99 (stable)	0.32	106.58	II
SpuHAK12	87.60	9.16	44.23 (unstable)	0.33	101.97	IV
SpuHAK13	64.65	8.84	35.23 (stable)	0.51	111.09	III
SpuHAK14	93.02	8.60	41.01 (unstable)	0.36	110.04	I
SpuHAK15	87.23	8.79	32.24 (stable)	0.188	104.11	III
SpuHAK16	87.88	6.53	41.87 (unstable)	0.38	107.77	I
SpuHAK17	83.50	9.05	35.12 (stable)	0.36	108.38	I
SpuHAK18	93.30	5.75	40.45 (unstable)	0.35	104.61	III
SpuHAK19	87.95	7.11	33.51 (stable)	0.39	114.42	II
SpuHAK20	91.56	7.93	32.10 (stable)	0.197	102.06	III
SpuHAK21	68.79	9.14	27.87 (stable)	0.53	113.34	III
SpuHAK22	91.43	8.55	37.08 (stable)	0.36	102.28	I

**Table 3:** Subcellular localization prediction of SpuKUP proteins*^a^*.

Gene	Plasma membrane	Mitochondrial inner membrane	Vesicles of secretory system	Vacuole membrane	Nucleus
*SpuHAK1*	66.7%	11.1%	11.1%	11.1%	—
*SpuHAK2*	66.7%	22.2%	—	—	11.1%
*SpuHAK3*	66.7%	11.1%	11.1%	11.1%	—
*SpuHAK4*	66.7%	11.1%	11.1%	11.1%	—
*SpuHAK5*	88.9%	—	11.1%	—	—
*SpuHAK6*	88.9%	—	11.1%	—	—
*SpuHAK7*	77.8%	22.2%	—	—	—
*SpuHAK8*	66.7%	11.1%	11.1%	11.1%	—
*SpuHAK9*	66.7%	22.2%	—	—	11.1%
*SpuHAK10*	77.8%	11.1%	11.1%	—	—
*SpuHAK11*	88.9%	—	11.1%	—	—
*SpuHAK12*	77.8%	11.1%	—	—	11.1%
*SpuHAK13*	66.7%	11.1%	11.1%	11.1%	—
*SpuHAK14*	66.7%	11.1%	11.1%	11.1%	—
*SpuHAK15*	66.7%	11.1%	11.1%	11.1%	—
*SpuHAK16*	77.8%	11.1%	—	11.1%	—
*SpuHAK17*	66.7%	11.1%	11.1%	11.1%	—
*SpuHAK18*	77.8%	11.1%	11.1%	—	
*SpuHAK19*	77.8%	11.1%	—	11.1%	—
*SpuHAK20*	66.7%	11.1%	11.1%	11.1%	—
*SpuHAK21*	77.8%	11.1%	11.1%	—	—
*SpuHAK22*	77.8%	11.1%	—	11.1%	—

^a^ indicates no detection.

**Table 4:** The *cis*-acting regulatory elements and numbers in the promoter regions of *SpuHAK* genes*^a^*.

Gene	Light	Anaerobic induction	Defense and stress	Drought-inducibility	Low temperature	Salicylic acid	Gibberellin	Methyl jasmonate	Abscisic acid	Auxin	Meristem expression	Endosperm expression	Circadian
*SpuHAK1*	10	3	2	—	—	1	1	4	—	1	—	—	—
*SpuHAK2*	12	1	—	—	—	—	1	—	2	1	1	—	—
*SpuHAK3*	15	2	2	2	1	—	—	6	7	2	1	—	—
*SpuHAK4*	5	2	—	—	—	—	—	—	1	—	—	—	1
*SpuHAK5*	5	3	—	2	—	1	—	4	1	1	2	—	—
*SpuHAK6*	15	3	2	—	—	2	—	—	4	—	1	1	—
*SpuHAK7*	8	4	—	1	—	1	—	4	—	—	—	3	—
*SpuHAK8*	8	4	—	1	1	—	—	—	—	2	—	—	—
*SpuHAK9*	5	2	—	1	—	—	1	8	1	2	1	—	—
*SpuHAK10*	9	3	2	—	—	—	3	2	4	—	—	—	—
*SpuHAK11*	12	3	1	—	—	2	—	2	1	—	1	1	1
*SpuHAK12*	12	4	—	—	—	—	1	4	2	3	1	—	—
*SpuHAK13*	10	2	1	1	—	—	1	2	3	—	1	—	1
*SpuHAK14*	9	1	—	—	—	2	—	—	—	1	—	—	1
*SpuHAK15*	11	2	—	—	—	—	—	2	1	—	1	1	—
*SpuHAK16*	15	1	2	1	—	—	3	6	4	1	1	—	—
*SpuHAK17*	12	2	2	—	1	—	—	4	3	—	—	—	1
*SpuHAK18*	9	1	—	—	—	—	—	2	—	—	—	1	—
*SpuHAK19*	17	3	—	1	1	—	1	4	7	—	1	—	—
*SpuHAK20*	14	2	—	1	—	—	—	2	5	—	—	1	—
*SpuHAK21*	9	4	1	1	1	—	1	2	—	2	1	—	—
*SpuHAK22*	10	2	—	2	2	2	1	6	3	—	—	—	—

^a^ indicates no detection.

## Data Availability

The data used to support the findings of this study are included within the article.

## Abbreviations

K^+^: Potassium
PEG: Polyethylene glycol
qRT-PCR: Quantitative real-time polymerase chain reaction.

## Supplementary Materials

Supplementary Materials Supplementary Table 1: specific primers used for quantitative RT-PCR.
