# Supplementary material for: Characterization and Expression of KT/HAK/KUP Transporter Family Genes in Willow under Potassium Deficiency, Drought, and Salt Stresses
Source: Biomed Res Int. 2020 Jun 9;2020:2690760. doi: 10.1155/2020/2690760 (PMC7303730; doi:10.1155/2020/2690760)
Supplement: Supplementary Materials — Supplementary Table 1: specific primers used for quantitative RT-PCR. [file 2690760.f1.doc]

Supplementary Table 1. Specific primers used for quantitative RT-PCR.

| Gene | Primer (5’ to 3’) | Amplicon size (bp) |
| --- | --- | --- |
| *SpuHAK1* | [F]: GTCCAGCATTTGCAGACCCA  [R]: CTCTGACCGGACGACCTAAC | 100 |
| *SpuHAK2* | [F]: GCTGCAAGAAGCTAACAGCAAA  [R]: GAGGCGACAAAGCATGAACAA | 215 |
| *SpuHAK3* | [F]: CAGAGCTGAGGGAGATCAAGT  [R]: TCCCTGGCATCCCTGTTTATCT | 104 |
| *SpuHAK4* | [F]: CGAAGGATGACTTGGAGGGAG  [R]: ACGGCGGTGACCTTATCTC | 150 |
| *SpuHAK5* | [F]: ACAGTCGACTAGCGTCTATGG  [R]: GCCGCAATCTACCGTGTTCT | 123 |
| *SpuHAK6* | [F]: TCTGTTCTTGTTTGTCCGGCT  [R]: TTGACCGGACGACCTACCT | 223 |
| *SpuHAK7* | [F]: GTCATAGGTGATGGCGTGCT  [R]: GCAAGCAAGACAATTACACCAC | 107 |
| *SpuHAK8* | [F]: GTTGCTCGACTGGCTGATTT  [R]: GCAAACCTATACTCACCCGA | 116 |
| *SpuHAK9* | [F]: TGGCAAGTCCATCAGGAGAT  [R]: CGGATAATCGCATTCTAGCACC | 102 |
| *SpuHAK10* | [F]: GTCGATGGAGTTACGGTGGG  [R]: TCGGAACAGCCTTTGCTCTG | 154 |
| *SpuHAK11* | [F]: TGTTCTTGTTTGTCCGGCTTG  [R]: TGACCGGACGACCTACCT | 220 |
| *SpuHAK12* | [F]: TGGCATAAGAGCCTATTTGGTG  [R]: AACCCATCCACCTTTGGGAA | 108 |
| *SpuHAK13* | [F]: CATCCCGCAGACTCGACA  [R]: CCTTGCCCTAATGTCTCCGT | 120 |
| *SpuHAK14* | [F]: GGGAATGATGAACCCAACTGC  [R]: TCAACGGCGGTGACCTTATCT | 175 |
| *SpuHAK15* | [F]: ATGGATCGAACTGCTGACATAG  [R]: GCCGATCAAGTGAACAACACC | 102 |
| *SpuHAK16* | [F]: GCAAGTGTGTCTGGCGGTTT  [R]: GGCTCATCATCAGCACCGA | 110 |
| *SpuHAK17* | [F]: GCCAGAGACTAGACGCAAGAG  [R]: CTAGTGCTGTTGACGGGCTT | 215 |
| *SpuHAK18* | [F]: AGATCAAAGACCGGAAGAACCA  [R]: CTCCAGACTTGGGTCTTCATCC | 100 |
| *SpuHAK19* | [F]: TTCCGTATGTTCCGTTGTGTTG  [R]: GTGATTCGGAAGAACATTTGGC | 210 |
| *SpuHAK20* | [F]: TGACGCTGAGCTTGGTATTT  [R]: TTAAGCATAGGCACCAGCACA | 169 |
| *SpuHAK21* | [F]: CCTTATGGCCACACCTCTACG  [R]: TTGGCTCTGAGCACGATGAA | 233 |
| *SpuHAK22* | [F]: TCGTTTAGGGAATTTGAAAGCACA  [R]: TTTCTGGACGCTCACTGC | 148 |
| *SpuActin* | [F]: TGGGTTTGCTGGAGATGAT  [R]: CAGTAGGAGAACTGGGTGC | 156 |
